# Supplementary material for: Patterns of Intron Gain and Loss in Fungi
Source: PLoS Biol. 2004 Nov 30;2(12):e422. doi: 10.1371/journal.pbio.0020422 (PMC532390; doi:10.1371/journal.pbio.0020422)
Supplement: Table S1 — Also available at http://genes.mit.edu/NielsenEtAl/. (4.3 MB ZIP). [file pbio.0020422.st001.zip › NielsenEtAl/html/1342.html]

AN1290.1.NCU07068.1.MG07661.1.FG02820.1


```
 CLUSTAL W (1.82) Multiple Sequence Alignments - Introns Inserted


Sequence 1: AN1290.1	883 aa
Sequence 2: NCU07068.1	932 aa
Sequence 3: FG02820.1	887 aa
Sequence 4: MG07661.1	930 aa
Alignment Length: 990 aa
Number Identitical Residues: 254 aa
Alignment Score (without introns) 13857


MG07661.1 	-------------------MSAATITAAPVATAASQAGIFEGMNPTVFNPSDPIVLFIIQ
NCU07068.1	MASAITDAVLKTVIRAVAATSSNTSSNATIPSVNGQAGVLEGANPIKYSASAPITLFIIQ
FG02820.1 	------------------MSSSPTTTAAAVTSAHPQGGIIEGLNPIVYNPKDPITLFIVQ
AN1290.1  	-------------------MADSSTENRAPP----QGGILEGGDPSQYDSKNPIVIFIIQ
          	                    :  :    . .    *.*::** :*  :... **.:**:*

MG07661.1 	~ATIVIGLTRLLYWPLAKIHEPKVIAEVIT0GIILGPSVMGRIPGFTAT~IFPPASMAPF
NCU07068.1	~AGIVIIFCQLLAYPLQWINQPKVIAEVIG~GILLGPSVMMRIPGFEAA1IFPKESMPVF
FG02820.1 	~AFVIIIFCRLLQWPLSKFGQPRVIAEVIG~GIVLGPSVMMRIPGFKEN~IFPTESMPVL
AN1290.1  	0AFIIIVLCRMLHWPLSKIRQPRVIAEVIA~GIILGPSVMGRVPNFTDS~IFPPESIPSL
          	 * ::* : ::* :**  : :*:******  **:****** *:*.*    ***  *:. :

MG07661.1 	RLAANIGLVLYLFLVGLEIDLSYLLRNWRIAVSVASFDMAIPFGLGVALAYGLYNEFKDE
NCU07068.1	NNVANLGLIIFLFLVALEVDMRMFTSNWKVALSVGLAGMILPFGLGFGIAWGLYHQFHND
FG02820.1 	SNVATIGLLLFLFLVGLEVDTRMFKSNWRVAVSVGLASMMLPFGLGVAVAWGLYEEYGSE
AN1290.1  	NLFANVGLVLFLFLVGLETNLRFLVSNWRVASSVSAAGMILPFGLGAGVSYGLYHEFHDE
          	   *.:**:::****.** :   :  **::* **.  .* :***** .:::***.:: .:

MG07661.1 	QGIAPISFGVYALFIGIAIAIT~AFPVLCRILTSLKLLNSNVGVIVLTSGIINDVVGWIL
NCU07068.1	GTTVPISFGVYGLFIGTALAIT~AFPVLCRILTELKLLRSNVGVTVLAAGIGNDVTGWIL
FG02820.1 	GTMKDMEFGTFALFIGTALAIT~AFPVLCRILSELQLLSTSVGVTVLAAGIGNDVVGWVL
AN1290.1  	EGLKPIDFGTYLLFIGIAMAIT0AFPVLCRILTELKLLGTNVGVIVLSAGVGNDVVGWIL
          	     :.**.: **** *:*** *********:.*:** :.*** **::*: ***.**:*

MG07661.1 	LALCVTLVNSGAGITALYILLVAVGFSLFLAYAVRPAFMWVLHKTHSLENGPTQGVVALT
NCU07068.1	LALCVALVNNNSGLAALWALLCCIGWILFLVFAVRPPFMWWVRRTGSLQNGPMQGVVALT
FG02820.1 	LALSVALVNNANGLTALYVFLTAVAWVLFLVYAVRPVFLWVLRRTDSIQNGPSQGITTLT
AN1290.1  	LALCVALVNAGSGLTALWVFLVCVGYVTFLVVIFRPLFLRFLNYTGSLQKGPSQSVVTIT
          	***.*:***   *::**: :* .:.:  **.  .** *:  :. * *:::** *.:.::*

MG07661.1 	IFMVLASAFFTS~IIGVHSIFGAFMVGLMCPHQGGFAVKLTEKIEDLVAVIFVPLFFALS
NCU07068.1	LLLVLFSAFFTN1IIGIHAIFGAFLVGLICPHEGGFAIKMTEKIEDLISVLFLPLYFALS
FG02820.1 	LLLVLASSWFTA1AIGVHAIFGAFLIGLICPHDGGFAIKLTEKIEDLVGSILLPLYFALS
AN1290.1  	ILIALASSFFTQ~VIGVHAIFGGFLIGLLCPHEGGFAIKLTEKIEDLVTALFLPLYFTLS
          	:::.* *::**   **:*:***.*::**:***:****:*:*******:  :::**:*:**

MG07661.1 	GINTNLGLLDSGRIWAYVFAVIFVAFFSKLIGGTIGARLNGLVWRESFTIGTLMSCKGLV
NCU07068.1	GLSTNLGLLNDGITWGYVIGVITCAFAGKIIGGTLAARANKLLWRESFTIGSLMSCKGLV
FG02820.1 	GLNTNLGLLDDGTTWGYVVAIIACAFFGKIIAGTLAARLTKCLWRESFTIGALMSCKGLV
AN1290.1  	GLQTDLGLLDTGIVWGYVVAVISIAFLAKVAGGALASRACGLLWRESFAIGTLMSCKGLV
          	*:.*:****: *  *.**..:*  ** .*: .*::.:*    :*****:**:********

MG07661.1 	ELIVL~NIGREARILSTRTFTIFVVMAL~VTTFLTSPLVMWLYPPSYQQKLELWKQGKID
NCU07068.1	ELIVL~NIGLQAKILSQRTFTIFVVMAL~VTTVATTPLTKALYPPWYQRKVERWRRGEID
FG02820.1 	ELIVL0NIGLQAGILSPRTFTMFVVMAV0VTTVTTSPLTRWLYPLSYRLKVEKWRRGEID
AN1290.1  	ELIVL0NIGLQAEILSHRTFTIFVVMAL~VTTFATTPLTTYIYPKWYQDKVDRWRRGEID
          	***** *** :* *** ****:*****: ***. *:**.  :**  *: *:: *::*:**

MG07661.1 	WDGNPIHPSEEEE--TGAAGALDKKDIATRLLVYIRADGLSSVLSTVSLFTSAGVQTESD
NCU07068.1	WDENPLVPNESGSNISDPSKPIGDQSSIQRMLVYLRLDSLPSLFAFITLLSPQAKPS-EV
FG02820.1 	WDGNPLQTEAQSS--EHKMEEALDKSQTNRLILHLRLDALPGLFNLVSLLGGSRKQTVPA
AN1290.1  	WDGTPIQPSDNNS-IAAATKEQLQSRQVRRLLAYLRLDGLSSICTLAALLSPNRPPTPKV
          	** .*: .. . .          ..    *:: ::* *.*..:    :*:      :   

MG07661.1 	SPAPFVDPNAAVHLNGESMKEGITPATATLMRPRPERLPLLRVHGYRLVELSERNSSVMQ
NCU07068.1	EPTDKGSNPCPPTRKQKK---------------------TLEVHGLRLIELTDRTSSVMQ
FG02820.1 	ITPTTNDVNADATPAEEKTQV-------------IPS-RPFEVRGVRLMELTDRTSSVMQ
AN1290.1  	HPEKAQSTKAPEAAAEESGAD-------------IQPDSSLQVHGIRLMELTDRDSSVMK
          	 .    .  .      :.                      :.*:* **:**::* ****:

MG07661.1 	VAD-------IEQYAAHDPIIKAFGTSAN----TASRDVVISGKIAVVPQNSFADTLADE
NCU07068.1	VTEGIAGEEEEELYSLRDPVINTFRTFASLSSPSMSNNVAVSGRVAIVPESSYAETLASY
FG02820.1 	SAE-------LDEFASRDAVFSAFQTFS------RLNGVAVAGQVSIIPTNAYAETIVKF
AN1290.1  	VSE-------VDEHSLWDPVINTFRAFG------QWHDISIMAGVSVVPEHSYADTVLGM
          	 ::        : .:  *.::.:* : .        ..: : . ::::*  ::*:*:   

MG07661.1 	AGNSRSDLILVPWSETGSISEVPSLYGGVP-----------STDPLCNDSFAQLATGIFD
NCU07068.1	AADTQSEFVLIPWSEYGSLTDWD---QPLSVLAGAAGANAGNDRFKGNVHLVEFMHKTLA
FG02820.1 	AEEARSDFMIIPWSTYGGIAEES---STAALIETG----NINDRFF-SRTYIDYVQNAIE
AN1290.1  	AREGTTDLLLIPWSETGALSEHQ---GGLDVDE--------RNRFA-NGPYTAFVSSILN
          	* :  ::::::***  *.:::                          .          : 

MG07661.1 	AAKNVAAVGVYLDRTLIAKSDAGVTETVAEGKRYQLTRHTTGISLVDTQPDRLAAKFFSA
NCU07068.1	EAERVCNAGILIDN-GFGGFGT---------CRTRVQGLLRSQHHHTSSQDQDQPTIIIK
FG02820.1 	RST--CTTGIFINR-VPHDVLT---------RKPTLTRTRTGLSVHSAHDGAVVQR--PV
AN1290.1  	ESS--CNVGVLVERSMYTRNAK---------ARPDLQRTLSARSVGSAMWVSP-----AA
          	 :   . .*: ::.:                 :  :     .     :            

MG07661.1 	EHRKKVIRVFFAGTEDDLFAVRLALQLSRSGSTELQVTVCYHHRDNGFIADFERLRAKIE
NCU07068.1	NRTHHVFLPFIGG-ADDRVALNLVLQLLGNNDNVTATVVHLKYTASTSNMK---------
FG02820.1 	DQRQIIFVPFIGG-KDDRAALLFALQLAHN-PHVSIHVVHLHFSEDDHEALNASPDSDPS
AN1290.1  	TRSHHIVLPFFGG-YDDRYALQFVLQLAQN-DQVTATIIHIELPVPTNQTSKVIAQPKQH
          	 : : :.  *:.*  **  *: :.***  .        :  .              ..  

MG07661.1 	QGPESGSVIFTTVEPQPAQPSVSEGEPSTGTTRGSDVLAALAGDEAHRNITTILIVGRTS
NCU07068.1	------------GEDEDTKLLATFRDSLPVEFASRVVFEESNLTEGKNDLVGTVLGLANV
FG02820.1 	TGNDLGKNAVDFPSASDMDLLSTAKNNAAGKLEGRVTFLEIPVDS-VRNLPDLAVAHARE
AN1290.1  	E-SRSDTQFDSFNPESDLVFFATLRDSLPDALSSRVVFKSVNPKD-KAAAVQIAIDAVKD
          	     ..        .      :  :  .    .  .:      .         :     

MG07661.1 	TLTSSEPS-EYFEVRSALGNVATRAAAEIRRCG--------------AKNISILVVQARK
NCU07068.1	TVGQQQKKKEKKGGGAGDVVVVGRRHAVVDSIEAIAGGSGAGAGGSNSSRVDVGGGSANG
FG02820.1 	LVGKSRYS-------VGDLIIAGRSHPLFD-------------------NLLVEDFGVER
AN1290.1  	EMAQSASK-------PGNVTVVGRRNNYVD------------------QTLDIDPASSDE
          	 : ..  .        .   :. *    .                   . : :       

MG07661.1 	VAVALPSTTSGPSGQTFERKASHESQASDIGEHAA--
NCU07068.1	GGLDLGRTVGVVAEGLLVTG-PVNASVLVVQAGGKRK
FG02820.1 	---DFQRTIGVLGDRFARAG--VDAGLLVIDDKQAKA
AN1290.1  	VSPEARWVLGAVAEAMVRTENRTVGNVLVLHAASRHN
          	 .     . .  .           .    :
```
